# Effects of Citicoline-Based Supplementation on Lipid Peroxidation Markers and Sirtuin-1 Expression in Ischemic Stroke

**DOI:** 10.3390/cimb48030314

**Published:** 2026-03-15

**Authors:** Todorka Sokrateva, Bogdan Roussev, Daniela V. Vankova, Deyana G. Vankova, Diana Ivanova, Mihael Tsalta-Mladenov, Darina Georgieva, Miglena N. Nikolova, Galya Mihaylova, Milka A. Nashar

**Affiliations:** 1 Department of Biochemistry, Molecular Medicine and Nutrigenomics, Faculty of Pharmacy, Medical University “Prof. Dr Paraskev Stoyanov”-Varna, 9002 Varna, Bulgaria; sokrateva@mu-varna.bg (T.S.); bogdanroussev@gmail.com (B.R.); daniela.vankova@mu-varna.bg (D.V.V.); deyana.vankova@mu-varna.bg (D.G.V.); divanova@mu-varna.bg (D.I.); miglena.todorova@mu-varna.bg (M.N.N.); galya.mihaylova@mu-varna.bg (G.M.); 2 Department of Neurology and Neuroscience, Faculty of Medicine, Medical University “Prof. Dr Paraskev Stoyanov”-Varna, 9002 Varna, Bulgaria; mihael.tsalta@mu-varna.bg; 3 Second Clinic of Neurology with ICU and Stroke Center, University Hospital “St. Marina”, 9010 Varna, Bulgaria; darina.georgieva@mu-varna.bg; 4 Department of Clinical Medical Sciences, Faculty of Dental Medicine, Medical University “Prof. Dr Paraskev Stoyanov”-Varna, 9002 Varna, Bulgaria

**Keywords:** ischemic stroke, oxidative stress, 8-iso-prostaglandin F_2_α, arachidonic acid, lipid peroxidation, citicoline, nutraceutical supplementation, Sirtuin-1, neuroprotection

## Abstract

Ischemic stroke (IS) is associated with pronounced oxidative stress and lipid peroxidation, which contribute to secondary neuronal damage. This study explored the effects of a six-month intervention with a new formulation containing citicoline, vitamin C, and extracts from green tea and aronia (Cytodeox™) on arachidonic acid (AA) metabolism, lipid peroxidation assessed by total 8-iso-prostaglandin F_2_α (8-iso-PGF_2_α), and Sirtuin-1 (*SIRT1*) expression in healthy controls (*n* = 43) and patients with IS (*n* = 53), both with and without comorbidities. AA and 8-iso-PGF_2_α were quantified in serum using UPLC–MS and ELISA, respectively, and the fold change in *SIRT1* expression was assessed in peripheral blood mononuclear cells (PBMCs) by RT-qPCR. In healthy controls, Cytodeox™ significantly lowered AA and 8-iso-PGF_2_α levels. IS patients showed markedly increased baseline 8-iso-PGF_2_α, indicating severe oxidative stress. Following supplementation, 8-iso-PGF_2_α levels increased in patients with comorbidities, particularly diabetes mellitus (DM), whereas an exploratory analysis suggested a decreasing trend in patients without comorbidities. *SIRT1* expression was significantly upregulated in IS patients, with the most pronounced increase observed in the DM subgroup, while remaining unchanged in controls. These findings suggest a protective, antioxidant, and membrane stabilising effect of Cytodeox™ under conditions of preserved or moderately impaired redox homeostasis, supporting its potential role as a preventive or early supportive intervention.

## 1. Introduction

Ischemic stroke (IS) is one of the leading causes of mortality and long-term disability worldwide. It is characterised by a sudden loss of blood flow to the brain, interrupting the supply of oxygen and nutrients and leading to rapid neuronal death [1]. IS results from multiple etiological mechanisms according to the TOAST classification, including large-artery atherosclerosis, cardioembolic sources, and small-vessel occlusive disease, which may remain asymptomatic for prolonged periods prior to clinical presentation [2]. In approximately one-third of cases, IS is caused by a buildup of atherosclerotic plaque, which may remain asymptomatic for years [3].

Understanding the molecular pathways involved in IS is crucial for developing effective therapeutic strategies. In this context, identifying a combination of biomarkers that respond to altered homeostasis during stroke is essential.

The pathological process of brain ischemia is associated with significant increases in free fatty acid (FFA) levels in human serum and cerebrospinal fluid (CSF) [4]. Higher FFA levels released during IS are associated with greater severity of ischemic injury [5]. Although the aetiology of their accumulation during ischemia remains unclear, it is suggested that the rapid rise in FFA levels results from impaired reacylation due to ATP depletion and from phospholipid deacylation due to increased activation of phospholipases [6]. The brain contains all three forms of phospholipase A2, and the cytosolic Ca^2+^-dependent PLA2 isoform (cPLA2) has a higher affinity for phospholipids that contain arachidonic acid (AA) [7]. AA, released during an ischemia-induced increase in intracellular calcium, is a precursor for leukotrienes, eicosanoids, and hydroxyeicosatetraenoic acid, which induce free-radical formation, compromise collateral flow, and attract leukocytes [8].

Oxidative stress plays a central role in the pathophysiology of cardiovascular, neurological, metabolic, and inflammatory diseases. IS is also characterised by increased formation of reactive oxygen species (ROS), leading to cell membrane damage and endothelial dysfunction. One of the most indicative markers of oxidative stress is prostaglandin F2α (8-iso-PGF_2_α) [9]. 8-iso-PGF_2_α, a representative of the F_2_-isoprostanes, is formed non-enzymatically upon peroxidation of AA and is considered a gold standard biomarker of in vivo lipid peroxidation [10]. Unlike classic prostaglandins, 8-iso-PGF_2_α, also known as 8-isoprostane, is formed primarily through the non-enzymatic, free-radical-catalysed peroxidation of AA in membrane phospholipids [11]. Via thromboxane A_2_ TP receptor signalling pathways (TP), 8-iso-PGF2α might be a potent vasoconstrictor contributing to endothelial dysfunction and an independent marker for cardiovascular disease [12,13]. In addition to its vascular effects, it has been shown to promote platelet activation and aggregation [14]. Accumulating evidence suggests that 8-iso-PGF_2_α is a biologically active lipid mediator—a molecular link between oxidative stress and chronic inflammation [15]. Elevated levels of 8-iso-PGF_2_α have been reported in atherosclerosis, diabetes mellitus, hypertension, cigarette smoking, and neurodegenerative disorders, highlighting its clinical importance in both cardiovascular and neurological pathology [16]. Furthermore, the plasma levels of 8-iso-PGF_2_α increase approximately 30-fold with ageing [17]. Overall, antioxidant supplements are the most consistently and statistically significantly associated with reductions in 8-iso-PGF_2_αrelative to baseline, except in chronic kidney disease, cystic fibrosis, and obesity [9].

Recent studies have highlighted the critical role of histone deacetylases, Sirtuins, in the pathophysiology of IS [18,19]. Sirtuins are a class of NAD-dependent histone deacetylases, divided into seven types based on localisation and physiological activities, and are recognised as silent information regulatory molecules [20]. Sirtuins exert a significant protective function by counteracting cellular stress and modulating metabolic processes during ischemia–reperfusion injury. Sirtuin-1 (SIRT1) is localised in both the cytoplasm and nucleus of mammalian cells and is expressed in many tissues, including the brain. It regulates cellular metabolism by modulating multiple downstream targets in response to stress [21]. Its influence extends to the regulation of energy metabolism, mitochondrial health, and genomic stability, making it a crucial target for potential therapies in neurological disorders [19,22]. Known to be widely expressed in key brain regions involved in metabolic regulation and neurodegeneration, and to have the potential to enhance cellular antioxidant defences, SIRT1 is essential for neuronal survival during cerebral ischemia [23].

Given the severe consequences of IS, including disability and reduced quality of life, extensive research has focused on supplementary therapies that address secondary injury processes and promote neurorepair [24].

Citicoline has been examined for its neuroprotective and restorative potential in both experimental models and clinical trials [25]. Citicoline is the generic name for cytidine 5′-diphosphocholine, an endogenous intermediate in the biosynthesis of phosphatidylcholine, a major phospholipid of cell membranes. Citicoline exerts neuroprotective effects through multiple mechanisms, including acting as a choline donor to enhance membrane repair and inhibiting phospholipid breakdown. By preventing the release of free fatty acids (FFA), citicoline may reduce oxidative stress and inflammatory signalling associated with the AA cascade [26]. Furthermore, experimental studies have shown that citicoline increases SIRT1 expression in the brain, which correlates with reduced stroke damage in rodent models, thereby demonstrating a key neuroprotective mechanism involving SIRT1 activation [27,28].

In addition to results demonstrating the effectiveness of citicoline supplementation in improving moderate cognitive impairment, studies have reported the combined administration of citicoline with other biologically active ingredients in recent years. The findings revealed enhanced therapeutic efficacy, attributed to additive effects [29,30].

Recently, a new supplement containing citicoline, combined with vitamin C, green tea, and aronia extracts, has been developed (Cytodeox™, Natstim Ltd., Sofia, Bulgaria). Although each ingredient has been extensively studied, the effects of this unique combination remain unclear.

Green tea (*Camellia sinensis*) and aronia (*Aronia melanocarpa*) extracts are rich in polyphenols, which are powerful antioxidants. Polyphenols mitigate ROS-induced lipid peroxidation and may protect endothelial and neuronal cells from oxidative injury, suggesting potential benefit in conditions characterised by oxidative stress, such as IS [31]. In addition, polyphenols activate SIRT1, thereby reducing oxidative stress and promoting cell survival [32].

Vitamin C (ascorbic acid) is a well-recognised antioxidant that contributes to redox homeostasis by directly neutralising ROS and regenerating other antioxidants [33,34]. Furthermore, it has been reported that it may significantly reduce levels of lipid peroxidation products, including 8-iso-PGF_2_α [35,36].

Despite advances in acute stroke care, neuroprotective strategies that mitigate secondary injury and enhance recovery remain limited. Elucidating the interplay among AA metabolism-driven neuroinflammation, the modulatory roles of SIRT1, and the therapeutic effects of citicoline can deepen understanding of ischemic pathology and potentially identify new targets for intervention.

This work aims to integrate current evidence on the relationship between IS, AA-mediated lipid signalling, *SIRT1* gene expression and the potential enhancement of neuroprotective outcomes through Cytodeox™ supplementation in healthy volunteers and patients with IS.

## 2. Materials and Methods

### 2.1. Participants

Of the initially screened 53 healthy volunteers, 3 did not meet the inclusion criteria and were excluded prior to enrolment. Specifically, two individuals were outside the predefined age limits, and one was excluded due to anticipated prolonged travel with uncertain availability for follow-up at the third month and blood sampling at the sixth month. Consequently, 50 participants were enrolled in the control group (CG). For the ischemic stroke patient group (ISP), 117 participants were recruited for this study, based on specific inclusion and exclusion criteria. The target recruitment size was approximately 50 healthy controls and 100 ischemic stroke patients, based on feasibility considerations and the expected patient flow in the clinic. This sample size was considered sufficient to detect moderate within-group changes in oxidative stress biomarkers using paired statistical analyses, which represented the primary outcome measures of the study.

The inclusion criteria for healthy volunteers were: aged between 40 and 75 years with no history of neurological comorbidities, including cerebrovascular disease, traumatic brain injury, neurodegenerative disorders, neuroinfections, or primary or secondary malignancies of the central nervous system. The exclusion criteria for this group were acute or chronic infectious or inflammatory diseases; pregnancy or lactation; transient ischemic attack; stroke within the previous 30 days; and dysphagia preventing tablet swallowing.

Eligible patients were those with acute IS who presented with acute focal neurological symptoms persisting for at least 24 h; had clinical evidence of neurological deficit; were hospitalized within 48 h of symptom onset; had neuroimaging study performed—computed tomography (CT) and/or magnetic resonance imaging (MRI) of the brain in the first 24 h of hospitalization, compatible with the clinical diagnosis of IS; had other causes of neurological symptoms excluded (hemorrhagic stroke, subarachnoid hemorrhage, processes occupying the intracranial space, neuroinfections, etc.); with severity of IS assessed by the National Institutes of Health Stroke Scale (NIHSS) ≤ 20 points; with absence of severe premorbid disability assessed by the Modified Rankin Scale (mRS) < 2 points; and had signed informed consent to participate in this study. Exclusion criteria were impaired consciousness; imaging evidence of primary or secondary central nervous system malignancies, hemorrhagic stroke, subarachnoid haemorrhage, or central nervous system trauma; prior (<3 months) or current treatment with citicoline-containing drugs; known allergy to any component of the study supplement; or withdrawal of informed consent.

### 2.2. Study Supplement

The supplement Cytodeox™ is a commercially available product (manufactured by Natstim Ltd., Sofia, Bulgaria) with ingredients per tablet comprising: 250 mg citicoline, 150 mg natural vitamin C derived from 70% standardised fruit extract from rosehip (*Rosa canina*), 100 mg leaf extract from green tea (*Camellia sinensis*), and 30 mg fruit extract from aronia (*Aronia melanocarpa*).

### 2.3. Study Design

This six-month intervention study involved two groups: healthy adult volunteers in the CG and ISP group. Healthy volunteers were recruited from March to June 2023, and the intervention took place from June to December 2023. Patients were recruited from among hospitalised individuals at the University Hospital “St. Marina”—Varna. The six-month intervention for patients, depending on their study enrolment, took place between October 2023 and October 2025. All subjects completed a questionnaire assessing their health status, lifestyle, medication use, and dietary supplement intake. Blood samples were collected before and after the intervention and distributed for various analyses. At baseline and at the third month, participants received their study supplement and were instructed to take 2 tablets daily after meals. After the intervention, all participants completed a questionnaire to assess therapy adherence and supplement tolerability.

The study adhered to the principles of the Declaration of Helsinki, and the study design, enrolment criteria, and all related documents were approved by the Research Ethics Committee of the Medical University of Varna (Protocols No. 119, 21 July 2022, and No. 140, 1 February 2024). Before entering the intervention, all participants provided written informed consent.

Of the 50 healthy volunteers enrolled, 43 (12 men and 31 women, mean age 52 ± 5.4) completed the intervention. Five participants withdrew due to illness, one due to gastrointestinal discomfort, and one for personal reasons. Among the 117 patients with IS, 53 (34 men and 19 women, mean age 63 ± 8.9) completed the intervention, resulting in a total of 96 participants.

During analyses, the ISP group was stratified into two subgroups: an IS with comorbidity group (ISC, *n* = 43) and an IS non-comorbidity group (ISN, *n* = 10). An additional subgroup from the ISC comprised patients with diabetic mellitus (DM).

**Figure 1:**
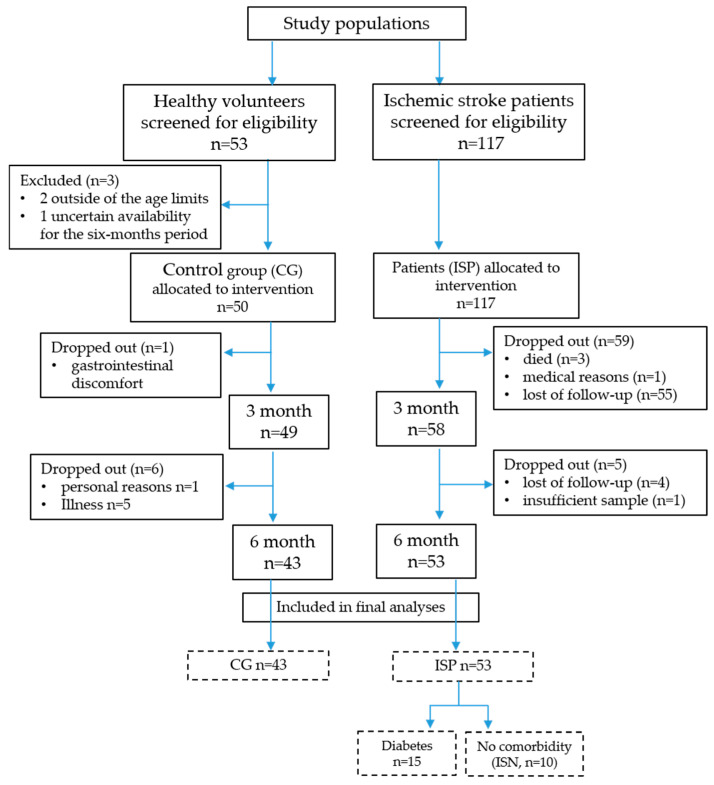
CONSORT 2010-adapted flow diagram of participant enrollment, allocation, follow-up, stratification, and analysis. CG—control group; ISP—ischemic stroke patient; ISN—ischemic stroke without comorbidity; The number of participants in the groups and subgroups included in the final analyses is presented in boxes with a dashed line.

Although the study was not randomised, participant flow and analysis were reported in accordance with the CONSORT 2010 framework to ensure transparency and reproducibility (Figure 1).

### 2.4. Biochemical Analyses

Lipid profile parameters, including total cholesterol (TC), HDL-cholesterol (HDL-C), LDL-cholesterol (LDL-C), and triglycerides (TAG), were analysed using an automatic biochemical analyser, Cobas 6000 (Hitachi High-Tech Corporation, Tokyo, Japan), in a certified clinical laboratory located at University Hospital St. Marina—Varna, Bulgaria. Total 8-iso-PGF_2_ (free and esterified isoprostane) was quantified using a competitive enzyme-linked immunosorbent assay (ELISA) with a commercial kit from Enzo Life Sciences AG (Enzo Life Sciences (ELS) AG—Lausen, Switzerland, Cat. No. ADI-900-091), in accordance with the manufacturer’s instructions. In the pre-analytical stage, the samples were subjected to hydrolysis to separate the membrane-bound 8-iso-PGF_2_α, in accordance with the manufacturer’s recommendations.

### 2.5. Determination of AA

The serum-free AA concentration was determined using a previously developed and validated in-house method that combined liquid–liquid extraction (LLE) with UPLC-MS analysis [37]. Briefly, 200 µL of serum was spiked with an internal standard (arachidonic acid-5,6,8,9,11,12,14,15-d8) and then subjected to LLE using a dichloromethane/methanol mixture (2:1, *v*/*v*) to precipitate proteins and maximise analyte recovery. The organic extracts were evaporated to dryness, reconstituted in the mobile phase, and filtered before analysis.

Chromatographic separation was performed on a Waters ACQUITY H-Class UPLC system with an AccQ-TAG Ultra C18 column (1.7 µm, 2.1 × 100 mm) maintained at 50 °C. Gradient elution was carried out using acetonitrile/isopropanol/water-based mobile phases containing ammonium formate and formic acid as additives. Detection was performed using a QDa single-quadrupole mass detector in negative electrospray ionisation (ESI−) mode. AA was quantified by Selected Ion Recording (SIR) at *m*/*z* 303.3, with peak identity confirmed by retention time and specific confirmation ions (*m*/*z* 304.3 and 259.3), compared to reference standards. The internal standard was monitored at *m*/*z* 311.32 Da (CV 20). Data analysis and processing were carried out using Empower^®^ 3.0 Chromatography Data Software (Waters Corporation—Milford, MA, USA).

The analytical method was fully validated in accordance with ICH M10 guidelines [38]. The method demonstrated excellent analytical performance with a limit of detection (LOD) of 0.046 µg/mL and a limit of quantification (LOQ) of 0.133 µg/mL, and showed good linearity over the concentration range of 0.5–5 µg/mL (R^2^ ≥ 0.998). Accuracy, expressed as percent recovery, ranged from 91.95% to 99.19%, while precision expressed as relative standard deviation (%RSD) ranged from 8.20 to 13.29% (intra-day) and 5.94–13.19% (inter-day). No significant carry-over or matrix interferences were observed. All measured serum AA concentrations in the present study were within the validated calibration range of the method. Detailed validation parameters, including recovery, calibration characteristics, matrix effects, and stability studies, are reported in the original methodological publication [37].

### 2.6. PBMC Isolation, mRNA Extraction and SIRT1 Gene Expression

Peripheral blood mononuclear cells (PBMCs) were isolated using Leucosep separation tubes from Greiner Bio-One GmbH, Frickenhausen, Germany (order No. 227290), following the manufacturer’s instructions. The isolated cells were resuspended in 250 mL PBS and transferred to sterile tubes. PrimezoleTM reagent (500 mL) from Canvax Biotech, S.L., Córdoba, Spain (Cat. No.: AN1100), was added for total RNA extraction. The protocol adhered fully to the manufacturer’s guidelines. Before proceeding to cDNA synthesis, the RNA quantification was measured using a multifunctional reader (Synergy 2, BioTek Instruments, Inc.—Winooski, VT, USA).

Extracted RNA (200 ng) was used for cDNA synthesis with the First Strand cDNA Synthesis Kit, which included oligo (dT)18 primer, reaction buffer, nuclease-free water, RNase inhibitor, nucleotide mix (dNTP), and reverse transcriptase. The reaction was performed according to the manufacturer’s instructions on the GeneAmp PCR System 9700 (Applied Biosystems—Foster City, CA, USA), with a final reaction volume of 20 μL. Following synthesis, each cDNA sample was diluted with 60 μL of molecular biology-grade water and stored at −20 °C.

For quantitative real-time polymerase chain reaction (RT-PCR), a preamplification step of 0.50 µL cDNA was used in a final reaction volume of 5 µL. The final primer concentration in the reaction mixture was 300 nm. Two-step RT-PCR analysis was performed with the AMPLIFYME SG Universal Mix kit (BLIRT S.A.—Gdańsk, Poland) using Rox Low fluorescent dye (BLIRT S.A.—Gdańsk, Poland). Reactions were run in 96-well plates with the following parameters: enzyme activation and denaturation at 95 °C for 3 min; denaturation at 95 °C for 5 s for 40 cycles; annealing at 60 °C for 10 s for 40 cycles; extension and fluorescence detection at 72 °C for 18 s for 40 cycles, followed by melting curve analysis. The analyses were performed on the QuantStudio 5 (Applied Biosystems). The fold change in gene expression due to intervention was calculated using the 2^−ΔΔCt^ method [38], and mRNA levels were presented as relative units (RU). The Actin beta (*ACTB*) gene was used as an internal control.

The nucleotide sequences of the genes were as follows (5′-3′): *ACTB* (F-GTG GCC GAG GAC TTT GAT TG; R-CCT GTA ACA ACG CAT CTC ATA), *SIRT1* (F-TAG ACA CGC TGG AAC AGG TTGC; R-CTC CTC GTA CAG CTT CACAGTC).

### 2.7. Statistical Analyses

Descriptive statistics were used to determine mean and median parameter levels and to assess the distribution of the groups and subgroups using the Kolmogorov–Smirnov normality test. A paired *t*-test was used in normally distributed groups to compare mean levels of the analysed parameters before and after the intervention. The Wilcoxon test was used to compare median parameter levels before and after Cytodeox™ supplementation in non-normally distributed groups. The Mann–Whitney U test was applied to analyse gene expression differences between groups with non-normal distribution data. An unpaired *t*-test was used to compare independent groups with normal distribution. A *p*-value below 0.05 was considered statistically significant. Statistical analyses were carried out using the software application GraphPad 6.

In addition, a post hoc statistical power analysis using Cohen’s method was performed for key outcomes in the ischemic stroke without comorbidity (ISN) subgroup using the observed effect sizes and a paired two-tailed design (α = 0.05). Power estimates were calculated to assess the study’s ability to detect changes in total cholesterol and 8-iso-PGF_2_α in this subgroup. These analyses were conducted to assess the robustness of subgroup findings and to guide the interpretation of exploratory analyses.

## 3. Results

### 3.1. Study Population and Group Distribution

A total of 170 individuals were initially assessed for eligibility, including 53 healthy volunteers and 117 patients with acute IS. After applying inclusion and exclusion criteria and accounting for dropouts during follow-up, 43 healthy volunteers and 53 ISP members completed the six-month Cytodeox™ supplementation and were included in the final analysis.

Among the ISP members, 10 individuals (18.8%) had no documented chronic comorbidities, whereas 43 patients (81.1%) presented with at least one major chronic condition, including arterial hypertension, diabetes mellitus, and/or dyslipidemia, stratified in ISN and ISC groups, respectively. Within the ISC group, 15 patients (28.3% of overall ISP group) had DM and were further analysed as a predefined metabolic risk subgroup.

### 3.2. Effects of Cytodeox™ on AA and Lipid Peroxidation in CG and ISPs

Baseline and post-intervention serum levels of AA and total 8-iso-PGF_2_α, a gold-standard marker of in vivo lipid peroxidation, are presented in Table 1.

In the control group, six-month Cytodeox™ supplementation led to a significant reduction in both oxidative stress markers. Total 8-iso-PGF_2_α levels decreased from 702.1 ± 484.6 pg/mL at baseline to 459.0 ± 376.0 pg/mL after intervention (*p* = 0.048). Similarly, serum AA concentrations declined substantially from a median of 3.09 μg/mL (interquartile range (IQR): 2.27–4.80) to 1.38 μg/mL (IQR: 1.05–1.98) (*p* < 0.0001), reflecting reduced mobilisation and oxidative turnover of membrane polyunsaturated fatty acids.

In contrast, patients with ischemic stroke exhibited notably higher baseline oxidative stress. Before supplementation, median 8-iso-PGF_2_α levels in the ISP group (12,594 pg/mL, IQR: 9079–16,129) were nearly twenty times higher than those in healthy controls (*p* < 0.0001), confirming significant systemic lipid peroxidation in the acute post-stroke phase. However, baseline AA concentrations did not differ significantly between the CG and ISP groups.

Following Cytodeox™ supplementation, total 8-iso-PGF_2_α levels in patients with ischemic stroke further increased to 13,911 pg/mL (IQR: 10,834–26,114; *p* = 0.0004 versus baseline), whereas changes in AA concentrations remained statistically insignificant (3.32 versus 3.52 μg/mL, *p* = ns). After the intervention, AA levels were significantly lower in the CG compared to the ISP group (*p* < 0.0001), indicating persistent activation of arachidonic acid-dependent oxidative and inflammatory pathways in stroke patients despite supplementation.

These divergent responses highlight a marked contrast between the effective attenuation of lipid peroxidation in healthy individuals and the persistence or progression of oxidative stress in the post-ischemic state.

**Figure 2:**
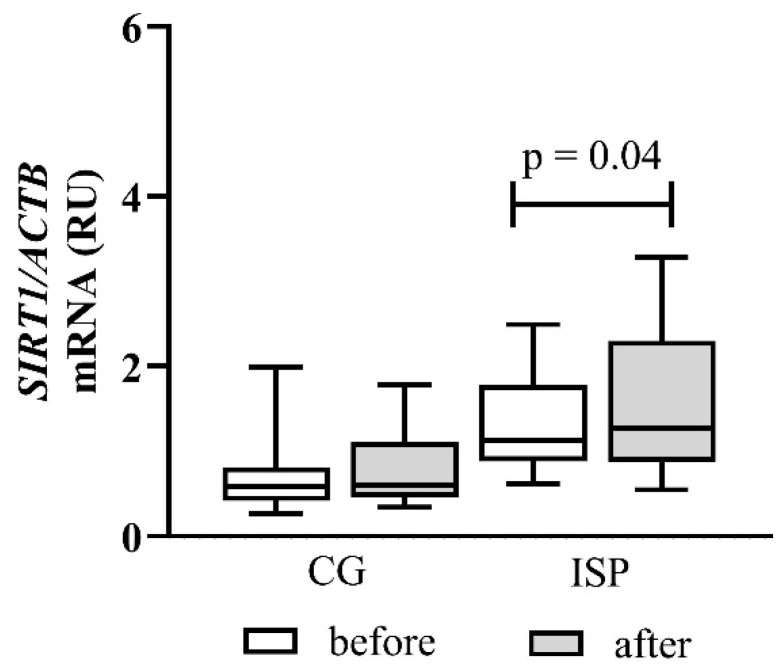
Effect of Cytodeox™ supplementation on *SIRT1* mRNA expression in PBMCs of CG (*n* = 43) and ISPs (*n* = 53). Analysis of relative gene expression was performed using 2^−ΔΔCt^ method. Data are presented as relative units (RU). The mean differences were analysed using Wilcoxon test. CG—control group; ISP—ischemic stroke patient.

Changes in *SIRT1* mRNA expression in peripheral blood mononuclear cells are presented in Figure 2.

At baseline, ischemic stroke patients showed a tendency toward higher *SIRT1* expression compared with healthy controls, although this difference did not reach statistical significance. After six months of Cytodeox™ supplementation, *SIRT1* gene expression remained unchanged in the CG, indicating that the intervention did not activate stress-response pathways under physiological conditions.

In contrast, a notable increase in SIRT1 expression was observed in the ISP group after supplementation (*p* = 0.04), indicating activation of natural cytoprotective and metabolic stress-response mechanisms in the post-ischemic state. This rise was consistent throughout the patient group and was independent of initial expression levels.

### 3.3. Cytodeox™ Effects in Ischemic Stroke with and Without Comorbidity

Baseline and post-supplementation lipid profile parameters in ISN patients are shown in Table 2. This subgroup was chosen for lipid profile analysis to prevent confounding effects from lipid-lowering pharmacotherapy, which was routinely prescribed for patients with comorbidities. None of them received statin or similar therapy.

At baseline, ISN patients had mildly elevated total cholesterol and LDL-C, while HDL-C and triglyceride concentrations were within reference ranges. After six months of Cytodeox™ supplementation, TC decreased significantly (*p* < 0.05), Cohen’s d = 0.85, post hoc power 68%. LDL-C showed a decreasing trend that did not reach statistical significance (Cohen’s d = 0.55, post hoc power 38%), whereas HDL-C decreased significantly compared with baseline (*p* < 0.05), Cohen’s d = 1.42, post hoc power 96%. Triglyceride concentrations remained unchanged throughout the intervention period.

**Figure 3:**
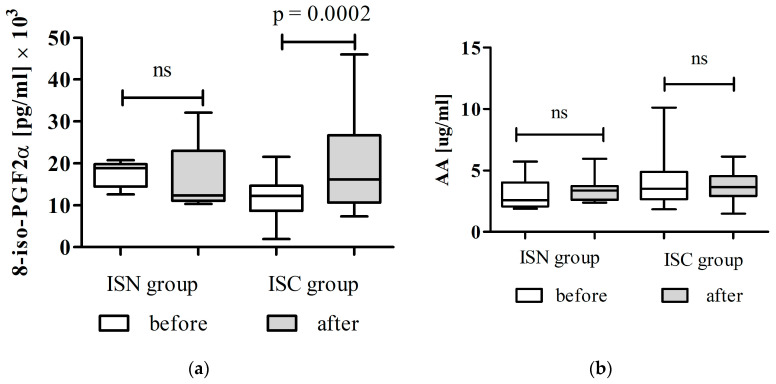
Effects of Cytodeox™ supplementation on oxidative stress markers in ISPs (*n* = 43) and ISN (*n* = 10): (**a**) total 8-iso-PGF_2_α and (**b**) AA. The Wilcoxon test was used to compare the levels of markers before and after the intervention. ISN—ischemic stroke patients without comorbidities; ISC—ischemic stroke patients with comorbidities. ns—no statistical significance.

Changes in oxidative stress markers among patients with ischemic stroke, stratified by comorbidity status, are shown in Figure 3a,b.

As shown in Figure 3a, total 8-iso-PGF_2_α levels decreased in ISN patients following Cytodeox™ supplementation, although the reduction was not statistically significant, with an effect size d = 0.77, and post hoc power about 10–15%. In contrast, the ISC group exhibited a significant increase in total 8-iso-PGF_2_α levels after supplementation compared with baseline (*p* < 0.0002), indicating persistent or worsened lipid peroxidation in this group.

Serum arachidonic acid concentrations are presented in Figure 3b. In ISN patients, AA levels showed a modest, non-significant decrease after supplementation, the effect size d = 0.53, and post hoc power 30%.

These results demonstrate a clear divergence in the oxidative stress response to Cytodeox™ supplementation, depending on baseline metabolic and inflammatory status. While patients without comorbidities exhibited a response pattern similar to that of healthy controls, those with comorbidities showed evidence of increased oxidative lipid metabolism despite supplementation.

### 3.4. Cytodeox™ Effects on Ischemic Stroke with Diabetes Comorbidity

**Figure 4:**
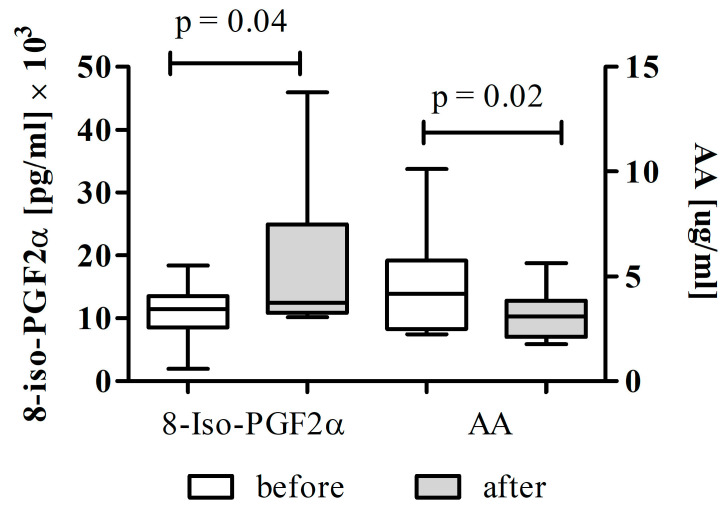
Effects of Cytodeox™ supplementation on oxidative stress markers in ISPs with DM (*n* = 15). The Wilcoxon test was used to compare total 8-iso-PGF_2_α and AA levels before and after the intervention.

To further elucidate the influence of metabolic dysregulation on supplementation efficacy, oxidative stress markers were analysed separately in patients with ischemic stroke and diabetes mellitus (DM subgroup). The results are shown in Figure 4.

In this subgroup, total 8-iso-PGF_2_α levels increased significantly following Cytodeox™ supplementation (*p* = 0.04), with modest statistical power (Cohen’s d = 0.61, post hoc power 60%) and the highest values observed across all study groups (Figure 4). In parallel, serum AA concentrations decreased significantly after intervention (*p* = 0.02, post hoc power 70%) (Figure 4).

This inverse relationship between AA and 8-iso-PGF_2_α suggests accelerated arachidonic acid utilisation as a substrate for oxidative lipid peroxidation in diabetic patients. The findings are consistent with chronic hyperglycemia-induced oxidative stress and impaired redox homeostasis characteristic of diabetes mellitus, which may override the antioxidant and membrane-stabilising effects of the nutraceutical intervention.

**Figure 5:**
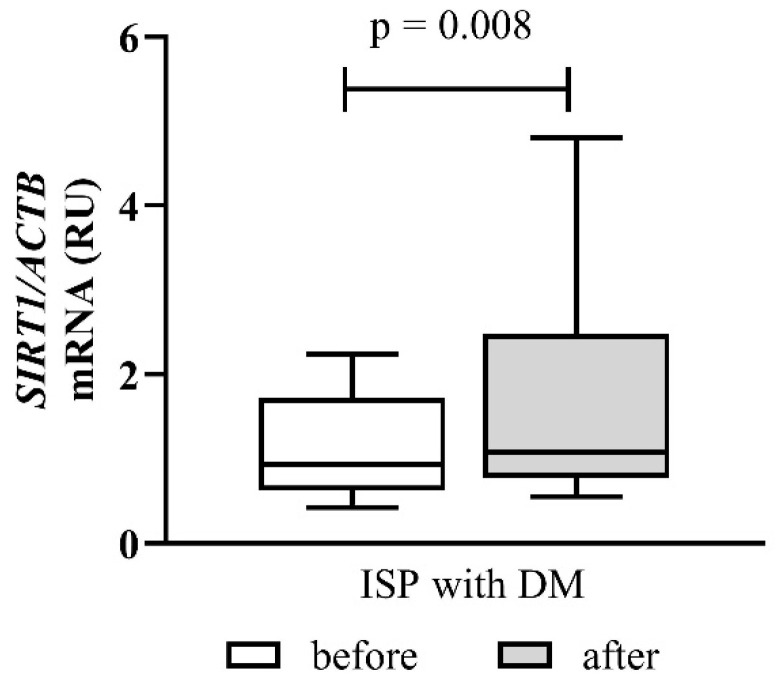
Effect of Cytodeox™ supplementation on *SIRT1* mRNA expression in ISPs with DM. The fold change in expression is presented as relative units (RU). Analysis of relative gene expression was performed using 2^−ΔΔCt^ method. Data are presented as relative units (RU). The mean differences were analysed using Wilcoxon test. ISPs—ischemic stroke patients; DM—diabetes mellitus.

Changes in *SIRT1* mRNA expression in ischemic stroke patients with diabetes mellitus are presented in Figure 5.

At baseline, diabetic patients showed higher *SIRT1* expression than healthy controls. After Cytodeox™ supplementation, *SIRT1* expression rose further in this subgroup, reaching statistical significance (*p* = 0.008, post hoc power ≈ 80–85%). The magnitude of *SIRT1* upregulation exceeded that observed in the overall ischemic stroke patient cohort.

This marked increase in *SIRT1* expression likely indicates the activation of compensatory stress-response pathways under combined ischemic and metabolic stress. Notably, *SIRT1* upregulation happened despite worsening oxidative stress markers, suggesting that increased *SIRT1* expression alone was insufficient to prevent ongoing lipid peroxidation in patients with diabetic ischemic stroke during the study period.

## 4. Discussion

The present study demonstrates that Cytodeox™, a combined formulation containing citicoline and antioxidant-rich plant extracts, has distinct biological effects that vary depending on the underlying redox and metabolic status of the population studied. The research involved healthy volunteers and patients with ischemic stroke, with and without comorbidities. The focus was on oxidative stress and lipid peroxidation, assessed using markers such as 8-iso-PGF_2_α, AA, and *SIRT1* gene expression.

Although participants were instructed not to change their dietary or lifestyle habits at the start of the study, these factors cannot be fully controlled in real-world settings. In this context, the non-randomised, open-label design without a placebo group introduces several potential sources of bias, including placebo effects and unmeasured confounding related to lifestyle modifications during the intervention period. At the end of the intervention, all participants reported full adherence to therapy, with no interruptions (as mentioned in Section 2.3). Additionally, from an ethical perspective, including a placebo group in patients with acute IS was considered challenging, given the potential neuroprotective benefit of the supplement and the vulnerability of the study population. Therefore, the study design prioritised patient safety and adherence to standard clinical care. To partially address these limitations, analyses were stratified by clinical subgroups, and the consistency of biomarker responses across groups was evaluated.

In patients with ischaemic stroke without comorbidities (ISN), Cytodeox™ supplementation significantly lowered TC, with accompanying decreases in LDL-C and HDL-C. Similar lipid-lowering effects have been previously reported in healthy volunteers receiving the same supplementation [39]. These changes align with well-established effects of green tea catechins, which reduce intestinal lipid absorption and hepatic cholesterol synthesis while limiting LDL oxidation [40,41,42]. Meta-analytic data indicate that green tea intake results in significant reductions in total and LDL cholesterol, with neutral or modest effects on HDL-C [43].

These findings suggest a modulatory effect of Cytodeox™ on lipid metabolism in patients with ischemic stroke who do not have chronic metabolic disorders, primarily evidenced by a reduction in total cholesterol. The accompanying decrease in HDL-C in the context of decreased TC should be interpreted cautiously. Reductions in HDL-C have been observed in antioxidant and polyphenol-based interventions and may indicate increased HDL turnover, enhanced reverse cholesterol transport, or changes in HDL subfraction composition rather than indicating adverse lipid alterations [44].

Vitamin C may also contribute by providing antioxidant protection against LDL oxidation, enhancing endothelial nitric oxide bioavailability, and reducing vascular inflammation [33,45]. Additionally, aronia polyphenols and anthocyanins are recognised to influence the expression of genes involved in intestinal cholesterol transport and efflux, thereby decreasing circulating cholesterol levels [46,47].

Assessment of lipid profile parameters in patients with ischemic stroke and comorbidities (ISC) was not feasible due to concurrent statin, fibrate, and antidiabetic medications, which exert dominant effects on lipid metabolism and would obscure supplement-specific outcomes. The interpretation of lipid-related outcomes in ISPs and comorbidities should also consider the influence of concomitant pharmacotherapy. Most ISC and DM patients received standard secondary prevention therapy, including statins, antihypertensives, and antidiabetic medications (Supplementary Table S1), which may mask potential lipid-modulating effects of Cytodeox™. Although a possible additive antioxidant interaction between Cytodeox™ and these therapies cannot be excluded, this hypothesis should be interpreted cautiously and requires confirmation in dedicated controlled studies.

A key finding of this study is the significant, parallel reduction in AA and total 8-iso-PGF_2_α in healthy volunteers following Cytodeox™ supplementation. Since 8-iso-PGF_2_α is formed predominantly through non-enzymatic, free-radical-mediated peroxidation of AA esterified in membrane phospholipids, its reduction reflects a genuine attenuation of lipid peroxidation rather than transient antioxidant scavenging [10,11]. The decrease in AA in healthy individuals should be interpreted as a marker of reduced mobilisation and oxidative turnover, rather than substrate depletion. Under conditions of low oxidative stress, AA remains largely esterified within membrane phospholipids and is not released for eicosanoid or isoprostane synthesis [48]. Citicoline likely contributes by enhancing phosphatidylcholine synthesis and membrane repair, thereby limiting phospholipase-mediated AA release [26,49]. Simultaneously, the antioxidant components of Cytodeox™ reduce the availability of reactive oxygen species, thereby decreasing the likelihood of non-enzymatic lipid peroxidation [31,35]. The reduction in 8-iso-PGF_2_α therefore reflects stabilisation of membrane redox status, consistent with previous findings of decreased malondialdehyde levels in healthy volunteers receiving the same supplementation [40].

Isoprostanes, such as 8-iso-PGF_2_α, are not merely biomarkers but biologically active mediators that promote vasoconstriction, platelet activation, and endothelial dysfunction through thromboxane A_2_ receptor signalling [14]. Elevated levels are associated with atherosclerosis, diabetes, ageing, and neurodegenerative diseases [17,50]. The marked reduction in 8-iso-PGF_2_α in healthy individuals therefore supports a protective and preventive role for Cytodeox™, potentially lowering baseline oxidative burden and reducing susceptibility to future vascular injury. These data suggest that the supplement acts primarily as a cytoprotective and membrane-stabilising agent, preserving phospholipid integrity under non-pathological conditions.

Compared with healthy volunteers, patients with ischaemic stroke exhibited persistently higher levels of oxidative stress. Total 8-iso-PGF_2_α increased significantly after supplementation, while AA levels remained stable across the entire ISP group. This indicates the overwhelming oxidative and inflammatory environment associated with cerebral ischaemia and reperfusion injury, further complicated by chronic conditions such as diabetes, hypertension, and dyslipidaemia [51,52].

The significantly higher 8-iso-PGF_2_α/AA ratio in patients with ischaemic stroke compared to controls indicates increased in situ lipid peroxidation rather than higher substrate availability [48]. Notably, patients with ischemic stroke without comorbidity showed a tendency towards lower 8-iso-PGF_2_α levels, similar to the response seen in healthy controls, suggesting that baseline metabolic health is a key factor in determining responsiveness to nutraceutical intervention.on.

The most notable oxidative response in the present study was observed in ISPs with DM comorbidity, where 8-iso-PGF_2_α increased significantly while AA levels decreased after Cytodeox™ supplementation. This pattern likely reflects the combined effect of chronic hyperglycaemia-induced oxidative stress and stroke-related activation of lipid-oxidation pathways. Diabetes mellitus is characterised by a significant imbalance between ROS production and antioxidant defence mechanisms, resulting in ongoing oxidative stress. Hyperglycaemia provokes mitochondrial overproduction of ROS and overwhelms endogenous antioxidant systems [53,54]. This mitochondrial dysfunction is a key mechanism underlying diabetic vascular complications [55]. Furthermore, patients with DM frequently present with additional cardiometabolic comorbidities such as dyslipidemia and hypertension, both of which further increase oxidative burden. In particular, dyslipidemia promotes oxidative modification of LDL, thereby accelerating atherosclerotic progression and amplifying ROS production within vascular tissues [56].

Alongside these diabetes-related mechanisms, IS itself activates PLA_2_ through calcium-dependent and inflammatory signalling pathways, leading to the release of AA from membrane phospholipids and increasing the pool of oxidisable lipid substrates [57]. When this enhanced availability of AA coincides with persistent ROS overproduction in diabetes, it notably promotes non-enzymatic lipid peroxidation and isoprostanes formation, such as 8-iso-PGF_2_α [58,59,60].

The observed inverse relationship between AA and 8-iso-PGF_2_α in the ISC/DM subgroup therefore likely reflects accelerated oxidative consumption of AA rather than effective membrane stabilisation. Under conditions of sustained oxidative pressure, AA is rapidly converted to isoprostanes through free-radical-mediated peroxidation, leading simultaneously to depletion of measurable AA and accumulation of stable lipid peroxidation end-products [61,62]. This substrate–product dynamic provides a mechanistic explanation for the increase in 8-iso-PGF_2_α despite the reduction in circulating AA concentrations.

Importantly, because the present study was observational and did not include a placebo-controlled arm, it cannot be determined whether 8-iso-PGF_2_α levels would have increased even further in the absence of Cytodeox™ supplementation. Therefore, a modest antioxidant effect of the formulation cannot be excluded, although its magnitude may have been insufficient to counteract the extremely high ROS burden characteristic of diabetic ischemic stroke.

In our study, *SIRT1* gene expression was notably higher in ISPs compared to healthy controls, aligning with reports that describe *SIRT1* upregulation as a natural response to hypoxia and oxidative stress [18,63]. Experimental studies demonstrate that SIRT1 promotes neuronal survival by deacetylating proteins involved in cell survival, such as p53, FOXO, and Smad7, thereby reducing apoptosis and inflammation [64,65,66]. Despite increased *SIRT1* transcription, some damaging factors may negatively affect enzyme activity. This could explain the observed dissociation between *SIRT1* gene expression and markers of oxidative stress. SIRT1 is an NAD^+^-dependent deacetylase that, during metabolic stress, faces activity limitations due to intracellular cofactor depletion [67]. Moreover, oxidative modifications may further impair the enzyme function. Prolonged hypoxia and metabolic stress have been associated with a biphasic SIRT1 response, characterised by an initial increase followed by a decline in function [68].

The absence of *SIRT1* induction in healthy volunteers indicates that supplementation does not unnecessarily activate stress-response pathways, further supporting its role as a preventive rather than a corrective intervention. Evidence of a biphasic SIRT1 response during prolonged hypoxia may explain why increased expression in stroke patients with ischaemia did not lead to a reduction in oxidative stress markers within the study period [69,70].

Cytodeox™ combines citicoline with vitamin C and polyphenol-rich plant extracts from green tea and aronia, which act through complementary mechanisms. Citicoline supports membrane phospholipid synthesis and limits arachidonic acid release, whereas vitamin C and polyphenols reduce oxidative stress and lipid peroxidation and may activate SIRT1-dependent protective pathways. In this context, a potential additive effect may be considered, although the present study did not directly compare Cytodeox™ with its separate constituents. Consequently, any potential additive or synergistic interactions should be interpreted cautiously and require confirmation in dedicated comparative studies.

Although the current findings highlight the preventive and cytoprotective role of Cytodeox™, the potential therapeutic benefits of citicoline-based interventions in the early post-stroke period should also be considered. Clinical evidence suggests that administering citicoline within the first 24–48 h after stroke onset may improve neurological recovery. In patients treated within 36 h of ischemic stroke, citicoline significantly enhanced cortical excitability parameters linked to neuroplasticity [71]. Similarly, patients receiving citicoline within 48 h after stroke showed greater improvements in neurological and functional scores compared to those with later initiation and prolonged therapy [72,73]. Dose may also influence therapeutic outcomes. A network meta-analysis of studies starting citicoline treatment within 72 h of stroke onset showed that both 500 mg and 2000 mg regimens improved functional outcomes and decreased mortality compared with controls, although the optimal dose remains undefined [30]. In addition to acute-phase benefits, longer-term citicoline therapy has been associated with improved cognitive outcomes and quality of life following stroke [74]. Mechanistically, citicoline may support neuroprotection by stabilising membrane phospholipids and increasing dopamine and norepinephrine levels in the central nervous system [75].

Together, these findings suggest that earlier initiation of Cytodeox™ supplementation after ischemic stroke may boost its neuroprotective potential, especially when combined with optimised dosing and adequate treatment duration.

## 5. Conclusions

Six-month Cytodeox™ supplementation lowered markers of lipid peroxidation, indicated by significant decreases in arachidonic acid mobilisation and 8-iso-PGF_2_α levels, suggesting improved redox balance and membrane stabilisation in healthy individuals and stroke patients without additional health conditions. Conversely, patients with ischemic stroke and metabolic comorbidities, especially diabetes mellitus, showed persistently elevated oxidative stress, implying that severe metabolic dysregulation may limit the effectiveness of the intervention. Overall, Cytodeox™ might serve as a preventative or early supportive strategy to maintain membrane integrity and minimise oxidative stress. Further controlled studies are needed to determine optimal dosing, earlier post-stroke application, and interactions with standard pharmacological treatments.

Several limitations of the present study should be acknowledged. First, the study employed a non-randomised, open-label design without a placebo-controlled arm. Although this approach was appropriate for an exploratory supplement intervention and reflects real-world clinical conditions, it limits causal inference and does not allow full control of placebo effects or unmeasured confounders. Beyond budget and time constraints, from an ethical point of view, including a placebo group could deprive patients with ischemic stroke of potentially beneficial supplementation.

Second, the analyses of oxidative stress biomarkers among patients with ischaemic stroke without comorbidity and in diabetes subgroups were based on relatively small numbers and should therefore be interpreted as exploratory. Larger, adequately powered studies are required to confirm these subgroup-specific trends.

Third, ischemic stroke patients with comorbidities were receiving standard pharmacological therapies, including statins, antihypertensives, and antidiabetic medications, which are known to influence lipid metabolism, oxidative stress, and inflammatory pathways. Although this reflects standard clinical practice, it may have masked or attenuated the potential effects of the supplement, particularly on lipid profile parameters. In addition, the study did not stratify ischemic stroke patients by etiological subtypes according to the TOAST classification, nor did it incorporate neuroimaging parameters such as infarct location, size, or vascular territory into the analysis. Although etiological classification according to the TOAST criteria was systematically recorded, the present study was designed as a mechanistic investigation of oxidative stress and was not statistically powered for subtype-specific analyses. Given that pathophysiological mechanisms and redox responses may differ among ischemic stroke subtypes, a dedicated etiological analysis incorporating TOAST stratification is warranted and will be addressed in a subsequent focused investigation of the cohort.

Finally, the dose and duration of the intervention were determined based on safety considerations, on the one hand, and on the financial and time constraints imposed by the project, on the other. It remains possible that higher doses, longer intervention periods, or earlier initiation after the onset of ischemic stroke may be required to achieve measurable reductions in oxidative stress in patients with severe metabolic dysregulation or advanced comorbidities.

Despite these limitations, the study provides valuable evidence of a preventive and cytoprotective effect of Cytodeox™ supplementation under conditions of preserved or moderately impaired redox homeostasis, and offers a rationale for future randomised, controlled trials in well-defined high-risk populations.

## Figures and Tables

**Table 1:** Serum arachidonic acid (AA) and total 8-iso-prostaglandin F_2_α (8-iso-PGF_2_α) levels in healthy controls (CG) and ischemic stroke patients (ISP) before and after six-month Cytodeox™ supplementation.

	8-iso-PGF2α (pg/mL)	*p*	AA (μg/mL)	*p*	Statistical Method
CG before vs. CG after Mean ± SD	702.1 ± 484.6 vs. 459.0 ± 376.0	0.048	3.09 _(2.270–4.800)_ vs. 1.38 _(1.050–1.980)_	<0.0001	Paired *t*-test/Wilcoxon test
ISPs before vs. ISPs after Median _(25–75%)_	12,594 _(9079–16,129)_ vs. 13,911 _(10,834–26,114)_	0.0004	3.32 _(2.53–4.53)_ vs. 3.52 _(2.78–4.26)_	ns	Wilcoxon test/Wilcoxon test
CG before vs. ISP before Mean ± SD/ Median _(25–75%)_	702.1 ± 484.6 vs. 12,377 ± 512	<0.0001	3.09 _(2.270–4.800)_ vs. 3.32 _(2.53–4.53)_	ns	unpaired *t*-test/Mann–Whitney test

ns—no statistical significance.

**Table 2:** Lipid profile parameters in ischemic stroke patients without comorbidities (ISN) before and after six-month Cytodeox™ supplementation.

Lipid Parameter	Before Intervention X ± SD	After Intervention X ± SD	*p*	Statistical Method
TC [mmol/L]	5.26 ± 0.86	4.63 ± 1.09	0.04	Paired *t*-test
LDL-C [mmol/L]	3.16 ± 1.16	2.62 ± 1.01	ns	Paired *t*-test
HDL-C [mmol/L]	1.52 ± 0.42	1.32 ± 0.40	0.005	Paired *t*-test
TAG [mmol/L]	1.27 ± 1.14	1.28 ± 0.46	ns	Wilcoxon test
TC/HDL-C	3.69 ± 1.07	3.63 ±1.27	ns	Paired *t*-test
LDL-C/HDL-C	2.20 ± 0.95	2.13 ± 1.04	ns	Paired *t*-test

Data are presented as mean ± standard deviation (X ± SD). ns—no statistical significance.

## Data Availability

The original contributions presented in this study are included in the article. Further inquiries can be directed to the corresponding author.

## Supplementary Materials

The following supporting information can be downloaded at: https://www.mdpi.com/article/10.3390/cimb48030314/s1.

## Abbreviations

The following abbreviations are used in this manuscript:

AA	Arachidonic acid
CG	Control group
CSF	Cerebrospinal fluid
DM	Diabetes melitus
ELISA	Enzyme-linked immunosorbent assay
ESI	Electrospray ionization
FFA	Free Fatty Acid
HDL-C	High-density lipoprotein cholesterol
IS	Ischemic stroke
ISC	Ischemic stroke comorbidity group
ISN	Ischemic stroke non-comorbidity group
ISP	Ischemic stroke patient
LDL-C	Low-density lipoprotein cholesterol
LLE	Liquid–liquid extraction
NF-κB	Nuclear factor kappa B
PBMCs	Peripheral blood mononuclear cells
PLA2	phospholipase A2
QDa	Quadrupole detector
ROS	Reactive oxygen species
SIR	Selected ion recording
*SIRT1*	Sirtuin-1
TAG	Triacylglyceride
TC	Total cholesterol
TP	Thromboxane A_2_ receptor
UCP1	Uncoupling Protein 1
UPLC-MS	Ultra-performance liquid chromatography–mass spectrometry
8-iso-PGF_2_α	8-iso-prostaglandin F_2_α
