# Supplementary material for: Effects of Citicoline-Based Supplementation on Lipid Peroxidation Markers and Sirtuin-1 Expression in Ischemic Stroke
Source: Curr Issues Mol Biol. 2026 Mar 15;48(3):314. doi: 10.3390/cimb48030314 (PMC13024967; doi:10.3390/cimb48030314)
Supplement: Supplementary file 1 [file cimb-48-00314-s001.zip › cimb-4164119-supplementary.pdf]

Supplementary Table S1. Comorbidities and post-hospitalisation pharmacological therapy in ISP included in the study (n = 53)

Patient subgroup	Number of patients (n)	Percentage of total (%)	Main comorbidity	Post-hospitalisation therapy
**Ischemic stroke without comorbidity (ISN)**	10	18.9	None reported	Standard secondary stroke prevention: aspirin protect
**Ischemic stroke with cardiovascular comorbidity**	28	52.8	Arterial hypertension, dyslipidemia, or other cardiovascular conditions	Antihypertensive medication and statin therapy
**Ischemic stroke with diabetes mellitus (DM)**	15	28.3	DM	Most patients received statins combined with aspirin as part of secondary stroke prevention. Concomitant antidiabetic therapy included oral antidiabetic agents and/or insulin
**Ischemic stroke with gastroesophageal reflux**	4	7.5	Gastroesophageal reflux disease	Proton pump inhibitors, in addition to standard stroke therapy
**Ischemic stroke with osteoporosis**	1	1.9	Osteoporosis	Anti-osteoporotic therapy in addition to standard stroke therapy

Additional details on therapy in diabetic patients: of the 15 patients with DM, 11 were treated with statins and aspirin after hospitalisation. Four diabetic patients were not treated with statins during the stroke period, with the following clinical histories:

One patient had previously received statin therapy before the ischemic stroke and continued with oral antidiabetic medication.

Two patients had been treated with statins prior to the stroke, one alongside oral antidiabetic therapy and the other with insulin therapy.

One patient received fibrates during the study period and had previously been managed with insulin therapy and a low-carbohydrate diet before the ischemic stroke.

Overall, the majority of stroke patients with cardio-metabolic comorbidities received antihypertensive therapy and lipid-lowering medication (primarily statins) as part of standard secondary stroke prevention. A low-fat diet was recommended to all patients upon discharge from hospital.
